# Healthcare-associated infections and antimicrobial use at a major referral hospital in Papua New Guinea: a point prevalence survey

**DOI:** 10.1016/j.lanwpc.2024.101120

**Published:** 2024-06-18

**Authors:** Stephanie J. Curtis, Roland Barnabas, Kelly A. Cairns, Donna Cameron, Benjamin Coghlan, Robert Jones, Jacklyn Joseph, Alu Kali, Dimitri Kep, Gemma Klintworth, Stephanie Levy, Matt Mason, Majella Norrie, Trisha Peel, Gilam Tamolsaian, Josephine Telenge, Nellie Tumu, Andrew J. Stewardson, Gabriella Ak, the COMBAT-AMR group, Benjamin Thomas, Cassius Maingu, Dellyne Polly, Hans Nogua, Jessica Mondowa, Joe Sokal, Josen Yem, Joyce Lawrence, Mathilda Rarah, Rose Olwont, Rupert Marcus, Saberina Silas, Stephanie Kialo-Davis, Alison Macintyre, Philip Russo, Rosaleen Kehoe

**Affiliations:** a Department of Infectious Diseases, The Alfred Hospital and School of Translational Medicine, Monash University, 85 Commercial Road, Melbourne, Australia; b Port Moresby General Hospital - 3 Mile, Taurama Road National Capital District, Port Moresby, Papua New Guinea; c Department of Pharmacy, Alfred Health, 55 Commercial Road, Melbourne, Australia; d Microbiological Diagnostic Unit Public Health Laboratory, The University of Melbourne at the Peter Doherty Institute for Infection & Immunity, 792 Elizabeth Street, Melbourne, Australia; e Health Emergencies Working Group, Burnet Institute, 85 Commercial Road, Melbourne, Australia; f School of Health, University of the Sunshine Coast, Sunshine Coast, 90 Sippy Downs Drive, Queensland, Australia

**Keywords:** Healthcare associated infection, Antimicrobial use, Infection prevention, Surveillance, Point prevalence study, Papua New Guinea

## Abstract

**Background:**

Healthcare-associated infections (HAI) and antimicrobial use (AMU) are drivers for antimicrobial resistance, and robust data are required to inform interventions and track changes. We aimed to estimate the prevalence of HAI and AMU at Port Moresby General Hospital (PMGH), the largest hospital in Papua New Guinea.

**Methods:**

We did a point prevalence survey (PPS) on HAI and AMU at PMGH in May 2023 using the European Centre for Disease Prevention and Control (ECDC) PPS protocol. We included all critical care patients and randomly sampled half of the patients in other acute-care wards. We calculated weighted HAI and AMU prevalence estimates to account for this sampling strategy. Weighted HAI estimates were also calculated for an expanded definition that included physician diagnosis.

**Findings:**

Of 361 patients surveyed in 18 wards, the ECDC protocol identified 28 HAIs in 26 patients, resulting in a weighted HAI prevalence of 6.7% (95% CI: 4.6, 9.8). Surgical site infections (9/28, 32%) were the most common HAI. When adding physician diagnosis to the ECDC definitions, more skin and soft tissue, respiratory, and bloodstream HAIs were detected, and the weighted HAI prevalence was 12.4% (95% CI: 9.4, 16.3). The prevalence of AMU was 66.5% (95%CI: 61.3, 71.2), and 73.2% (263/359) of antibiotics were from the World Health Organization Access group.

**Interpretation:**

This is the first reported hospital PPS of HAI and AMU in Papua New Guinea. These results can be used to prioritise interventions, and as a baseline against which future point prevalence surveys can be compared.

**Funding:**

Australian Government 10.13039/501100000996Department of Foreign Affairs and Trade and Therapeutic Guidelines Limited Australia.

**Research in context**

**Evidence before this study**

There are currently no data on the prevalence or incidence of healthcare-associated infections and antimicrobial use in Papua New Guinea. Standardised point prevalence survey protocols, such as the European Centre for Disease Prevention and Control protocol, have been used across a range of settings. Despite their widespread use, these protocols may not be suitable in all settings due to their reliance on pathology, microbiology, or radiology investigations for diagnosis of healthcare-associated infections.

**Added value of this study**

According to the European Centre for Disease Prevention and Control protocol, there was a low prevalence of healthcare-associated infections at a major referral hospital in Papua New Guinea compared to similar settings. The prevalence of antimicrobial use was consistent with similar settings. When physician diagnosis of a healthcare-associated infection was added to the European Centre for Disease Prevention and Control definitions, the prevalence of healthcare-associated infections almost doubled. The true prevalence of healthcare-associated infections likely falls between the two estimates.

**Implications of all the available evidence**

We have provided the first prevalence estimates of healthcare-associated infections and antimicrobial use in Papua New Guinea. These estimates provide a useful baseline understanding and can be used to select and prioritise targeted interventions that prevent healthcare-associated infections and inappropriate antimicrobial use. Standardised surveillance protocols need to be suitable to the setting to allow for robust data collection.

## Introduction

Healthcare-associated infections (HAI) and antimicrobial resistance (AMR) are major, interrelated, threats to global human health. HAIs can contribute to AMR by resulting in increased antibiotic selection pressure, whilst extended hospital stays can increase the likelihood of patients acquiring a drug-resistant pathogen, and both HAIs and AMR require strong infection prevention and control measures to mitigate their threat. The recent World Health Organization (WHO) *Global strategy on infection prevention and control* aims to ensure everyone accessing or providing healthcare is safe from associated infections by 2030.^1^ Progress toward this aim to tackle HAIs and AMR requires implementation of HAI and antimicrobial use (AMU) surveillance to plan and evaluate quality improvement strategies.^2^ There are, however, limited HAI and AMU data available from low- and middle-income countries, including the Pacific region.^3^^,^^4^ In Papua New Guinea, data are limited to few small-scale studies in patient sub-populations collected without the use of standardised protocols that are not representative of HAI and AMU at the facility-level.^5^, ^6^, ^7^, ^8^ Robust hospital-level data are critical for selecting and prioritising the most appropriate interventions to prevent HAIs and avoid inappropriate AMU.^2^

Hospital surveillance data can be collected using a range of methodologies. A point prevalence survey (PPS) provides a snapshot in time. It is less resource intensive than continuous, prospective surveillance, and can be repeated to provide information on progress over time.^9^ In 2011, the European Centre for Disease Prevention and Control (ECDC) developed a standardised PPS protocol to assess HAIs and AMU, which has subsequently been revised and updated.^10^ The ECDC protocol has been used globally in both high and low-and middle-income settings, however, it may not be suitable in all settings due to their reliance on medical investigations (pathology, microbiology, radiology) for HAI diagnosis that may not be available or consistently applied.^1^^,^^3^^,^^9^^,^^11^, ^12^, ^13^, ^14^, ^15^, ^16^, ^17^

Given the absence of data in Papua New Guinea, we sought to provide the first prevalence estimates of HAI and AMU at the country's principal referral hospital, Port Moresby General Hospital (PMGH). Our core objectives were to: (i) estimate the prevalence of HAIs and describe the types of HAI; (ii) estimate the prevalence of AMU and describe the most frequently used antimicrobials; and (iii) describe the infection prevention control infrastructure at the hospital. Our secondary objective was to assess the suitability of the ECDC protocol in a setting where pathology, microbiology and radiology investigations are used infrequently compared to high-income settings.

## Methods

### Setting

We conducted a PPS of HAIs and AMU at the PMGH in Papua New Guinea from 16 to 19 May 2023. PMGH is the largest healthcare facility in Papua New Guinea with a catchment area of over 700,000 people, that also acts as a referral facility for provinces throughout the country. It is a teaching hospital, with 1200 beds, including a 7-bed intensive care unit, and provides medical, surgical, obstetrics and gynaecology, and paediatric care. Most wards have a ‘Nightingale’ design, with a large room and four rows of beds that are not divided by walls or curtains. Some wards have an isolation room available. The infection prevention program is governed by an active infection prevention committee, with an infection prevention team comprising of two full-time equivalent nursing staff and five short-term contract nurses. At the time of this project, there were no local antibiotic therapeutic guidelines.

### Study design

The PPS protocol was adapted from the ECDC approach (Version 5.3 2016).^10^ Points of variation from the ECDC protocol are listed in Supplementary Material 1.

### Eligible wards and participants

All acute care inpatient wards were included, excluding the emergency department, psychiatry and rehabilitation wards. Patients were eligible for inclusion if they were admitted to an eligible ward prior to 8:00 am on the day of the survey and had not been discharged from the ward by the time the survey was conducted. Patients absent from the ward at the time of the survey were excluded.

### Sampling methodology

We included all eligible patients on high-acuity wards, including the critical care ward, Cardiothoracic Unit and Intensive Care Unit. For feasibility, we utilised a random sampling methodology for patients in other eligible wards sampling half of all inpatients. Sampling occurred at ward level, with each ward allocated at random to inclusion of ‘odd’ or ‘even’ beds. All eligible patients in the corresponding beds were included. Where a bed was empty or the patient's record was not available, we sampled the subsequent adjacent bed, but thereafter returned to the dedicated odd or even sequence. Randomisation was performed using Microsoft Excel™ (Version 2311).

### Capacity building

This study was performed as part of COMBAT-AMR, a project funded by the Australian Government Department of Foreign Affairs and Trade to improve responses to AMR across the Pacific. Training of the infection prevention committee, infection prevention team and study data collectors in Papua New Guinea consisted of an introductory two-day workshop on HAI surveillance (October 2022) and a one-day workshop on PPS immediately prior to data collection.

### Data collection

During data collection, four teams were active each day of the PPS and team composition rotated daily. Each team included at least three members; a PMGH infection control nurse, a PMGH physician, and a COMBAT-AMR project team member (epidemiologist, infection control consultant, and an infectious diseases physician, all of whom had experience with HAI surveillance). Where possible, the PMGH physician collected data on the ward where they usually worked to minimise the study impact on clinical care. The team structure was designed to build local capacity, illustrate how research can be incorporated into routine care, and standardise the approach to data collection. Handheld tablet devices were used to enter data into the REDCap electronic data capture tools hosted at Monash University.^18^^,^^19^

For each ward surveyed, we collected ward- and patient-level data. Ward-level data related to infection prevention and control infrastructure, including the number of alcohol-based handrub dispensers, clinical handbasins (and an assessment of whether these were functioning with both soap and running water available), the number of single rooms available on the ward and the number of patients subject to transmission-based precautions. We also collected data on bed capacity and occupancy. Ward-level data was collected once, on the day of the patient data collection.

Patient-level data included age, sex, medical specialty, admission date, presence of invasive device, drug allergy documentation, and AMU data. AMU data included agent, route of administration, indication for treatment, and anatomical site of infection. Antimicrobial indication was determined by the data collection team through a review of the patient notes and discussion with the treating physician if documentation of the indication for treatment was unclear. The indication was classified as community infection, hospital infection, surgical prophylaxis, medical prophylaxis, or unknown. For the PPS, HAIs were active infections acquired by patients during their stay in a hospital or another healthcare setting, defined according to ECDC definitions.^10^ An infection was considered active, when new or acutely worse signs/symptoms of the infection were present on the survey date, or the signs/symptoms were present in the past and the patient was receiving treatment for that infection on the survey date.^10^ Patients underwent a complete chart assessment to detect HAIs, according to ECDC definitions, if either of the following ‘triggers’ were present: documented fever >38 °C within the last 24 h or current prescription of one or more antimicrobials where the patient was not being treated for a community infection. If a team was uncertain about how to interpret documented data, they consulted with another data collection team to reach a consensus.

### Statistical analysis

The point prevalence of HAIs was estimated from the proportion of patients in the sample with one or more HAI and the point prevalence of AMU was estimated from the proportion of patients in the sample administered at least one antimicrobial at the time of the survey. To account for the sampling approach, we used inverse probability weighting to calculate HAI and AMU point prevalence estimates. We weighted each participant by the number of inpatients on the ward divided by number of surveyed inpatients on that ward. We also estimated the point prevalence of HAIs through an ‘expanded’ HAI definition that combined infections identified by the ECDC criteria (as above) with physician diagnosis of an HAI. All estimates were computed with 95% confidence intervals (CI) using the Clopper-Pearson method. All data were analysed using R version 3.6.2.

### Ethical considerations

Ethics approval, with a waiver of individual consent, and participation of all international investigators, was provided by the PMGH Ethics Committee [EC#014], the Papua New Guinea Medical Research Advisory Committee [MRAC#23.18], and Alfred Health Human Research Ethics Committee [HREC#23/296]. A waiver of individual consent was approved as the data collected were non-identifiable, there was no interaction between researchers and patients, and there was no alteration to the usual care of patients. In addition, the work of COMBAT-AMR was performed within the context of a multi-institutional governance agreement involving all participating organisations, which was submitted as part of the ethics approval process.

### Role of the funding source

This work was funded by Therapeutic Guidelines Limited Australia, and the Australian Government Department of Foreign Affairs and Trade's Indo-Pacific Centre for Health Security funded COMBAT-AMR Program. The funders had no role in study design, data collection, analysis, manuscript preparation or the decision to publish.

## Results

### Patient demographics

The PPS included 361 eligible patients in 18 wards. The median number of patient sampled per ward was 19 patients [IQR: 16–24]. Patients were more frequently female (59.3%, 214/361) and they had a median age of 27 years [interquartile range (IQR): 13–40 years, range 0–86 years]. Most patients were admitted to hospital via the emergency department (81.4%, 294/361), and were under the care of medical (31.0%, 112/361), surgical (24.9%, 90/361), obstetrics and gynaecology (22.2%, 80/361) and paediatric (21.9%, 79/361) units. A high proportion of patients had invasive devices including peripheral vascular catheters (59.8%, 216/361) and indwelling urinary catheters (23.8%, 86/361). The median hospital length of stay at the time of the PPS was six days [IQR 3–17 days, range 0–207 days]. Patient characteristics are detailed in Table 1.

**Table 1:** Characteristics of patients in the point prevalence survey, by patients with and without a healthcare associated infection.

	All patients, n (%)	Patients without a HAI, n (%)	Patients with a HAI, n (%)
**Patients**	361	335	26
**Female sex**	214 (59.3)	204 (60.9)	10 (38.5)
**Age,** median years [IQR]	27 [13, 40]	27 [14, 40]	29 [9, 42]
**Age**			
Neonate^a^	25 (7.0)	22 (6.6)	3 (11.5)
0–14	72 (20.2)	67 (20.2)	5 (19.2)
15–29	102 (28.6)	96 (29.0)	6 (23.1)
30–44	83 (23.2)	77 (23.3)	6 (23.1)
45–59	44 (12.3)	42 (12.7)	2 (7.7)
60+	31 (8.7)	27 (8.2)	4 (15.4)
**Medical specialty**			
General Medicine	79 (21.9)	72 (21.5)	7 (26.9)
Paediatrics	79 (21.9)	72 (21.5)	7 (26.9)
Obstetrics & Gynaecology	80 (22.2)	78 (23.3)	2 (7.7)
General Surgery	26 (7.2)	21 (6.3)	5 (19.2)
Orthopaedic Surgery	28 (7.8)	28 (8.4)	0 (0.0)
Neurosurgery	12 (3.3)	11 (3.3)	1 (3.8)
Ear, Nose and Throat Surgery	11 (3.0)	10 (3.0)	1 (3.8)
Oncology	8 (2.2)	7 (2.1)	1 (3.8)
Other medical speciality	25 (6.9)	24 (7.2)	1 (3.8)
Other surgery speciality	13 (3.6)	12 (3.6)	1 (3.8)
**Emergency admission**	294 (81.4)	271 (80.9)	23 (88.5)
**Length of stay prior to the PPS,** median days [IQR]	6 [3, 17]	6 [2, 14]	19 [12, 28]
**Documented fever in last 24 h**	33 (9.2)	24 (7.2)	16 (61.5)
**Presence of invasive device**			
Peripheral venous cannula^b^	216 (59.8)	195 (58.2)	21 (80.8)
Central venous catheter^b^	10 (2.8)	7 (2.1)	3 (11.5)
Invasive ventilation^b^	9 (2.5)	7 (2.1)	2 (7.7)
Non-invasive ventilation^b^	14 (3.9)	13 (3.9)	1 (3.8)
Urinary catheter^c^	86 (23.8)	75 (22.4)	11 (42.3)

IQR, interquartile range; HAI, healthcare associated infection; PPS, point prevalence survey.

a A child in the first 28 days of life.

b Past 48 h.

c Past 7 days.

### Infection prevention and control infrastructure

At the time of the PPS, the bed occupancy across the surveyed wards was 89.5% (654/731). The bed occupancy was lowest on the private ward (10/29, 34.5%) and two obstetrics and gynaecology wards were above bed capacity at 107.6% (99/92) and 122.9% (43/35). There were a total 205 functioning alcohol-based handrub dispensers, with a mean ratio of functioning alcohol-based handrub dispensers to open beds of 1:4, which was highest in critical care wards, 1:1. There were 87 clinical handbasins with a ratio of 1 clinical handbasin for every 8 open beds. However, only 42.5% (37/87) had both running water and soap reducing the functioning clinical handbasins to open beds ratio to 1:20. Across the surveyed wards at the time of the PPS, three patients (0.8%) were in transmission-based precautions; two in contact precautions and one in airborne precautions.

### Antimicrobial usage

Of 361 patients, 242 were receiving antimicrobial therapy at the time of the survey, a crude AMU point prevalence of 67.0% (95% CI: 62.0, 71.7) and a weighted AMU point prevalence of 66.5% (95% CI: 61.3, 71.2). Community-acquired infection was the most common indication for an antimicrobial prescription (51.1%, 225/440). Prolonged (>24 h) antimicrobial courses were observed in 43.2% (16/37) of surgical prophylaxis prescriptions. Over half of antimicrobial agents were administered via the parenteral route (55.2%, 243/440). Antimicrobial drug allergy status was documented in the current drug chart of 10.3% (25/242) of patients. The most frequently prescribed antimicrobials were ceftriaxone, metronidazole and amoxicillin, followed by fixed dose combination antituberculosis therapy. Antimicrobial usage details are outlined in Table 2.

**Table 2:** Antimicrobial use at Port Moresby General Hospital at the time of the point prevalence survey, May 2023.

	Total, n (%)
**Patient-level characteristics** (n = 361)	
**Patients receiving antimicrobial therapy**	242 (67.3)^a^
Medical wards (*n* = *4*)	68/85 (80.0)
Surgical wards (*n* = *4*)	66/99 (66.7)
Obstetrics & gynaecology wards (*n* = *3*)	41/90 (45.6)
Paediatric wards (*n* = *4*)	57/71 (80.3)
Critical care wards (*n* = *3*)	10/16 (62.5)
**Antimicrobial agents prescribed per patient,** median agents [IQR]	1 [0, 2]
**Antimicrobial drug allergy box completed on current drug chart**	25 (10.4)
**Antimicrobial-level characteristics** (n = 440)	
**Length of antimicrobials prior to PPS**^b^, median days [IQR]	3 [2–6]
**Most common antimicrobial agents**	
Ceftriaxone	63 (14.3)
Metronidazole	59 (13.4)
Amoxicillin	52 (11.8)
Isoniazid + Rifampin + Pyrazinamide + Ethambutol	50 (11.4)
Flucloxacillin	43 (9.8)
Benzylpenicillin	31 (7.1)
Gentamicin	30 (6.8)
Sulfamethoxazole-trimethoprim	24 (5.4)
Other agents	88 (19.9)
**World Health Organization AWaRe classificatioñ**	
Access group	263 (73.2)
Watch group	93 (25.9)
Reserve group	2 (0.6)
Not classified	1 (0.3)
**Route of administration**	
Parenteral	243 (55.2)
Enteral	194 (44.1)
Topical	3 (0.7)
**Indication**	
Treatment of community acquired infection	225 (51.2)
Medical prophylaxis	77 (17.5)
Treatment of healthcare associated infection	67 (15.2)
Surgical prophylaxis	37 (8.4)
Unknown	30 (6.8)
Other	4 (0.9)
**Duration of antimicrobials with an indication of surgical prophylaxis**	
0–1 day duration	21 (56.8)
>1 day duration	16 (43.2)
**Site of infection**^c^	
Respiratory tract	111 (37.5)
Skin, soft tissue or deep decubitus ulcer	55 (18.6)
Gastrointestinal tract	42 (14.2)
Central nervous system	22 (7.4)
Bloodstream	15 (5.1)
Bone, joint, bursa, or spinal disc	13 (4.4)
Eye, ear, nose, throat or mouth	12 (4.1)
Other	11 (3.7)
Urinary tract	8 (2.7)
Reproductive tract	5 (1.7)
Cardiovascular system	2 (0.7)

IQR, interquartile range; CI, confidence interval.

a The medication chart for one patient was unavailable but ongoing receipt of antimicrobials was clearly documented in medical notes.

b Excludes antituberculosis therapy.

c Only for infections with an indication of community-acquired or healthcare-associated infection; ∼Only for antibiotics, n = 360.

### Healthcare-associated infections

**Fig. 1:**
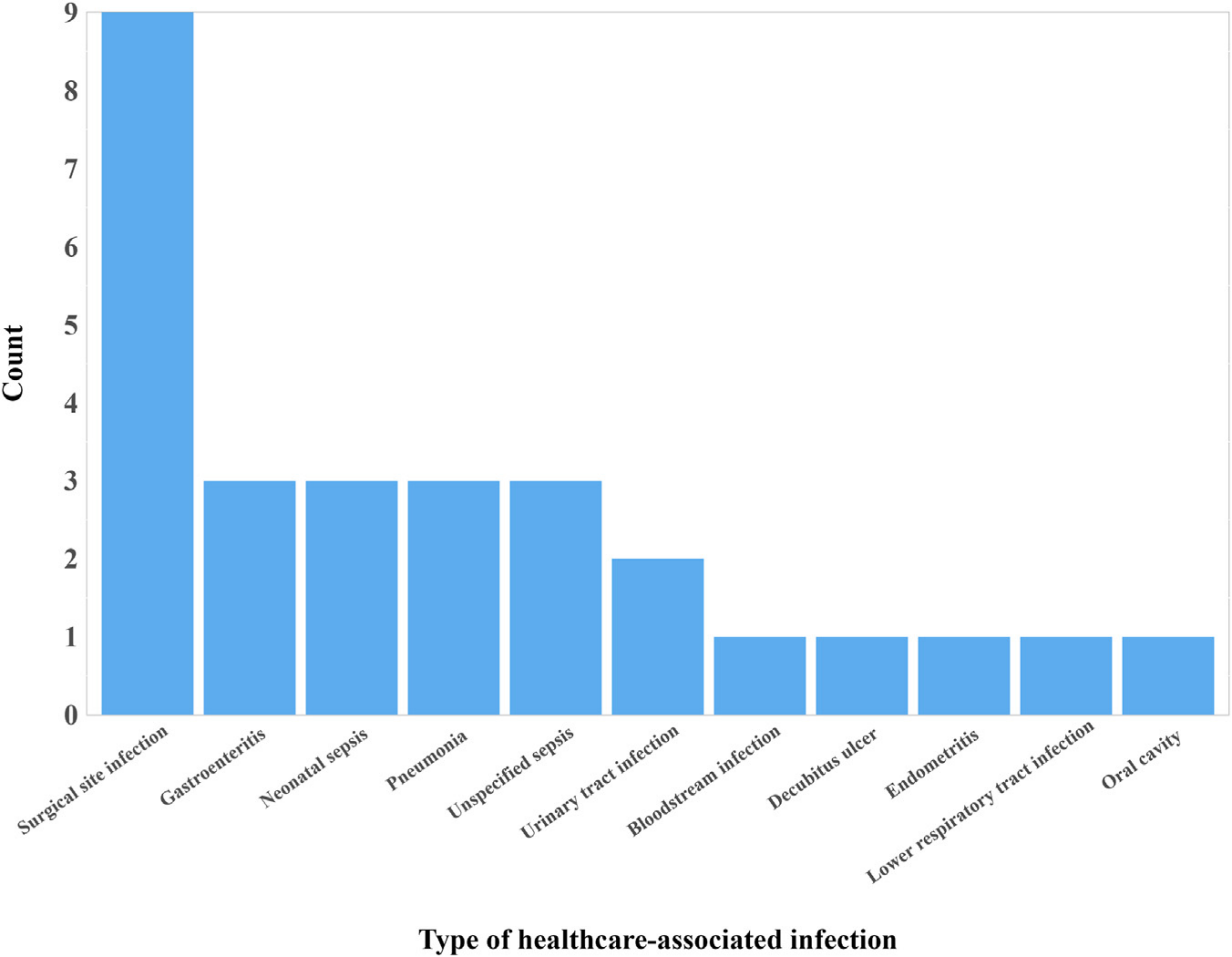
**Healthcare-associated infections at port moresby general hospital identified by the European Centre for Disease Prevention and Control point prevalence survey protocol**.

There were 28 HAIs in 26 patients; a crude point prevalence of one or more HAI of 7.2% (95% CI: 4.9, 10.3), and weighted point prevalence of one or more HAI of 6.7% (95% CI: 4.6, 9.8). All but one of the HAIs originated from PMGH (96.4%, 27/28), with the other attributed to an infection from another hospital (3.6%). The most common HAI identified was surgical site infections (32.1%, 9/28), and these occurred following nine distinct surgical procedures. Fig. 1 presents the HAIs identified. Of the three cases of pneumonia identified, one was ventilator-associated. Of the two urinary tract infections identified, one was catheter-associated.

Pathogens were identified for 14.3% (4/28) of HAIs. A total of seven microorganisms were reported amongst those four HAIs: an extended-spectrum-β-lactamase (ESBL)-producing *Klebsiella pneumoniae* was isolated from a patient with an organ space surgical site infection. An ESBL-producing *Serratia marcescens* was isolated from blood culture in a patient with a lower respiratory tract infection. *Proteus mirabilis* (antimicrobial sensitivity unknown) and other bacteria not listed were isolated from the patient with a bloodstream infection. Methicillin-sensitive *Staphylococcus aureus,* and *Escherichia coli* and *P. mirabilis,* both sensitive to ceftriaxone, or ceftazidime and carbapenems, were isolated from the patients with an infected decubitus ulcer.

When combining antimicrobial prescription with a physician diagnosis of a HAI with the ECDC HAI definitions, 47 patients had one or more HAI identified, a weighted HAI point prevalence of 12.4% (95% CI: 9.4, 16.3). Of the additional 21 patients with a physician diagnosis of a HAI, the site of infection was skin, soft tissue or deep decubitus ulcer (*n* = *11*), respiratory tract (*n* = 5), bloodstream (*n* = 3), gastrointestinal tract (*n* = *1*), urinary tract (*n* = *1*) and eye, ear, nose, throat or mouth (*n* = 1).

## Discussion

In our PPS at the largest healthcare facility in Papua New Guinea, the weighted HAI point prevalence was 6.7% according to the ECDC protocol. This almost doubled, to 12.4%, when we expanded the definition to include physician diagnosis of an HAI. Antimicrobial usage was common, with two thirds of patients administered one or more antimicrobial. Alcohol-based handrub was frequently available across all surveyed wards, however clinical handbasins for handwashing were frequently not functional.

The prevalence of HAIs in our study was slightly lower than reported in similar settings. In low-and middle-income countries the WHO estimated a pooled prevalence of HAI to be 15.5%, although the prevalence ranged from 7.1% to 22.2% when the ECDC criteria was applied.^3^^,^^12^, ^13^, ^14^, ^15^, ^16^, ^17^^,^^20^ The variation in international rates of HAIs may reflect a real difference in the risk of HAIs across settings or differences in healthcare systems, such as the type of facilities sampled. It may also be due to limitations in the suitability of the protocol outside of high-income settings. Surveillance protocols with standardised definitions allow for comparisons in infection rates across settings and over time at the facility level, however current protocols are designed for primary application in high-income-countries and may not be suitable in low-and middle-income countries due to the less frequent utilisation of pathology, microbiology and radiology investigations which are commonly required to fulfil criteria to diagnose a HAI.^3^^,^^14^^,^^21^

In PMGH, a pathogen was identified in only a minority of patients with HAIs and there were few respiratory infections suggesting that less frequent use of laboratory and radiological investigations could be a contributing factor in the low prevalence of HAIs estimated using the ECDC criteria. In contrast, the prevalence of HAIs almost doubled when the definition was expanded to include a physician's opinion. With this approach, we detected more respiratory, skin and soft tissue, and gastrointestinal infections, all of which typically rely on medical investigations to support the definition of an HAI under the ECDC protocol. This higher figure is likely an overestimate; it is expected that the true prevalence falls between our two estimates. Standardised HAI definitions that consider the practice of medicine and the use of medical investigations in low resource settings are required. The WHO is developing HAI definitions that are more appropriate for low-and middle-income countries; these will need to be consistently applied across facilities and countries to track trends and the impact of measures to prevent and control HAIs.^22^

In our study, surgical site infections were the most common HAI, accounting for one third of HAIs identified. Similar findings have been observed in Fiji, Ghana, Malawi, Nigeria and Pakistan, with surgical site infections accounting for 32–40% of all HAIs identified by the ECDC criteria.^3^^,^^13^, ^14^, ^15^, ^16^ The ECDC criteria allow for physician diagnoses of surgical site infections, which may explain why these infections comprise of the majority of HAIs in resource poor settings. Whilst the distribution of HAIs across medical specialties were, overall, proportionate to the number of patients admitted under that medical specialty, physician diagnoses of surgical site infections may also explain the slightly higher proportion of HAIs relative to the number of patients admitted in the medical specialty of general surgery. At the ward level one medical and one surgical ward had a higher prevalence of HAIs than the other wards their medical speciality. While this may be due to random variation (given the low numbers within each ward), it presents an opportunity for review and potential targeted intervention. The frequency of other types of HAI are more variably reported across jurisdictions and likely relate to the availability of high-quality diagnostic services and the underlying disease burden.^3^^,^^13^, ^14^, ^15^, ^16^ For instance, a significant share of the burden of infectious diseases in Papua New Guinea is attributable to neonatal sepsis and gastroenteritis, and PMGH receives both paediatric and adult patients from across the country; we found these to be important causes of HAIs.^23^^,^^24^ In contrast, healthcare-associated bloodstream infections are more common elsewhere, which may reflect underutilisation of blood cultures in Papua New Guinea.^12^, ^13^, ^14^, ^15^, ^16^

Antimicrobial usage was common, with two thirds of patients administered one or more antimicrobial, a finding comparable to other acute-care settings in low-and middle-income countries.^3^^,^^12^, ^13^, ^14^^,^^25^ AMU was similar across all medical specialties, with the exception of slightly lower use in critical care patients which may be a chance finding due to relatively low due to relatively low patient numbers and the lowest use in obstetrics and gynaecology patients, likely due to shorter admission for pregnancy without the need for antimicrobials. We identified that 43% of patients with an antimicrobial indication of surgical prophylaxis were receiving the antimicrobial for longer than 24 h. This is lower than in similar settings and may represent an undocumented but valid clinician concern for infection or inappropriate AMU.^3^^,^^25^ Nine of ten patients surveyed did not have the drug allergy box completed on their current medication chart, which may be due to a lack of hospital policy, or awareness of existing policy. This represents opportunity for improved antimicrobial stewardship and patient safety. Third generation cephalosporins (mainly ceftriaxone) and penicillins, were frequently used, whilst fluoroquinolone and carbapenem use was infrequent. Fluoroquinolones are widely used globally, but cost, access or physician familiarity could preclude their use at PMGH.^26^ Low carbapenem use could reflect differences in availability, local patterns of antimicrobial resistance or existing antimicrobial stewardship efforts. The WHO categorise antimicrobials into three groups to assist assessments of appropriate use, which includes a 60% target for “antibiotics that have a narrow spectrum of activity and a good safety profile in terms of side-effects” (‘Access’ group classification).^27^ At PMGH this targeted was exceeded; almost three quarters of antibiotics prescribed were in the ‘Access’ group. This target was achieved by 15 out of 19 reporting countries in 2020.^28^

Alcohol-based handrub was frequently available across all wards surveyed, with the highest availability in critical care wards. Since 2020, alcohol-based handrub has been produced on site at PMGH using the WHO methodology in response to the COVID-19 pandemic.^29^ While there are no recommendations for the number of alcohol-based handrub dispensers in health facilities, in our study, PMGH did not meet the WHO recommendations for functional clinical handbasins of a minimum of one sink to ten beds.^30^ This is critical gap in PMGH's hospital infection prevention and control infrastructure, which has been highlighted as a core objective of the National Action Plan on antimicrobial resistance in Papua New Guinea. An additional core objective of the National Action Plan is to strengthen surveillance, diagnostic capacity, and research on antimicrobial resistance, which remains a gap at PMGH due to limitations in trained microbiologists and laboratory facilities for timely culture and antimicrobial susceptibility testing.

This study has some limitations. First, this was a single-site study at a major tertiary referral hospital, and estimates are not reflective of HAI and AMU in other health facilities across Papua New Guinea. Second, a single PPS may limit temporal generalizability at a health facility, however this can be improved through a pragmatic approach of repeated PPS over time. Third, we used a pragmatic sampling method to reduce the burden on PMGH staff and we did not perform formal competency assessments or examine interrater reliability for data collection. However, we performed computer-generated random sampling to reduce selection bias and rotated teams to improve consistency in data collection and reduce information bias. Fourth, for feasibility, we did not explore risk factors for HAI and AMU, however the objective of this study was to provide the first prevalence estimates in Papua New Guinea. Finally, we did not perform an assessment of antimicrobial appropriateness as this assessment is resource intensive and often relies on local antibiotic guidelines, which were not available at the time of the PPS but have since been finalised.

This study documents the first prevalence estimates of HAI and AMU using a standardised protocol in Papua New Guinea. We report a low rate of HAIs compared to similar settings that also used the ECDC protocol, however this may be an underestimation due to the protocol's reliance on diagnostic investigations for HAI diagnosis which are used less frequently at PMGH. We found that PMGH had an equivalent prevalence of AMU to similar settings, and successfully met the WHO target for the proportion of antibiotics in the ‘Access’ group. Effective interventions to reduce the risk of HAI and improve antimicrobial stewardship require robust and regular data collection systems using protocols that are suitable for and sustainable in Papua New Guinea., Finally, we described the infection prevention control infrastructure at PMGH which has identified several avenues for improvement, all of which are consistent with the Papua New Guinea National Action Plan on Antimicrobial Resistance.

## Contributors

Conceptualization: SC, BC, AS, GA; Data curation: SC Formal Analysis: SC; Funding acquisition: BC, TP, AS; Investigation: SC, KA, RB, DC, RJ, RK, DK, MN, NT, AS, GA; Methodology: SC, KC, TP, AS, GA; Training: SC, KC, DC, GK, RK, MM, TP, AS; Project administration: SC, GK, SL, GT, AS; Supervision: AS, GA; Visualisation: SC; Writing—original draft: SC, AS, GA; Writing—review & editing: all authors. Access to raw data and data verification: SC, AS. Final responsibility for the decision to submit for publication: AS, GA.

## Data sharing statement

The data that support the findings of this study are available from the corresponding author, SJC, upon reasonable request, subject to approval by the relevant institutional review boards.

## Declaration of interests

There are none to declare relevant to this work. TP is the recipient of a Medical Research Future Fund grant [GNT2014635] and is a Board Director for the Australasian Clinical Trial Alliance. KC receives royalties from UpToDate as co-author of chapter “Daptomycin: An overview”, and their workplace has been paid Honoraria for presentations.

## Appendix A Supplementary data

Supplemental Data

Study Group
