# Supplementary material for: Healthcare-associated infections and antimicrobial use at a major referral hospital in Papua New Guinea: a point prevalence survey
Source: Lancet Reg Health West Pac. 2024 Jun 18;48:101120. doi: 10.1016/j.lanwpc.2024.101120 (PMC11238180; doi:10.1016/j.lanwpc.2024.101120)
Supplement: Supplemental Data [file mmc1.docx]

**Supplementary Material 1. Summary of major differences in study protocol compared to European Centre for Diseases Control (ECDC) protocol.**

| **ECDC protocol** | **Deviations** | **Rationale** |
| --- | --- | --- |
| **Sampling methodology** | | |
| - All patients admitted to the ward before or at 8:00 am and not discharged from the ward at the time of survey, including neonates on maternity and paediatric wards, will be included. | - All patients in high-acuity wards and 50% of patients in non-high acuity wards sampled admitted to the ward before or at 8:00 am and not discharged from the ward at the time of survey, including neonates on maternity and paediatric wards, will be included. | - Insufficient resources to sample every patient. |
| **Data Collection Processes** | | |
| - Composition of the team responsible for data collection varied from one hospital to another | - Data collectors included physicians and infection prevention control nurses from Port Moresby General Hospital (PMGH) and visiting data collectors that had facilitated the data collection training. All staff were trained in using the tool. Data collection occurred in rotating teams of three or four team members, usually including an infection prevention control nurse from PMGH, a physician from PMGH and a visiting data collector. | - To minimise the burden of time commitment required by the PMGH staff, as regular work duties were required to continue. - To build capacity of local staff to conduct healthcare-associated infection (HAI) surveillance. - Rotation of local staff across teams helped improve consistency of HAI reporting. |
| **Patient Data Fields** | | |
| - McCabe score was employed to classify the severity of underlying medical conditions. | - No risk factor data was collected, and trigger questions were used for efficient data collection. Patients with either a fever within the last 24 hours or a current antimicrobial prescription with an unknown indication or documented HAI, underwent a complete chart assessment to determine whether a patient had a HAI, according to the ECDC protocol. | - Insufficient resources to collect risk factor data and work through the HAI branching logic of the data collection for every patient. |
| **Antimicrobial Usage Data Fields** | | |
| - Anatomical Therapeutic Chemical (ATC) code noted for all antimicrobials, | - No ATC code as collected; however, the names of all antimicrobials were collected. | - Reduced data collection time. - ATC codes can be linked retrospectively if required. |
| **HAI Algorithm Data Fields** | | |
| - Patient has documentation in the medical record of any laboratory confirmation test for COVID-19 (viral RNA target or antigenic detection from an oropharyngeal or nasal swab or any other appropriate clinical specimen) and, Patient has no signs or symptoms compatible with mild/moderate COVID-19, or Patient has any sign or symptom compatible with COVID-19*, without need for oxygen therapy and oxygen saturation level ≥ 92%, or Patient has signs or symptoms compatible with COVID-19* with need for oxygen therapy for shortness of breath due to COVID-19 and/or oxygen saturation level <92% | - No COVID-19 (SARS-CoV-2 infection) was assessed. | - COVID-19 infections are not investigated or documented and no SARS-CoV-2 testing is performed at PMGH. |
| **Data Validation** | | |
| - Recommended sample size at the national level was 750 patients in 25 hospitals. | - The sample size was determined pragmatically by the number of eligible patients during the study. | - This was a single site study. However, data on total eligible inpatients were collected to allow for weighted prevalence estimates. |
| - Validation team was separate from the original data collection team and recommended to be blinded. | - Data collection teams involved rotating teams of physicians and infection prevention control nurses from PMGH and a visiting data collector. | - Prioritisation of building capacity of local staff to perform ongoing PPS through providing training and data collection practice to many PMGH staff members. However, the use of a rotating data collection roster improved data consistency. |
