# Supplementary material for: Healthcare-associated infections and antimicrobial use at a major referral hospital in Papua New Guinea: a point prevalence survey
Source: Lancet Reg Health West Pac. 2024 Jun 18;48:101120. doi: 10.1016/j.lanwpc.2024.101120 (PMC11238180; doi:10.1016/j.lanwpc.2024.101120)
Supplement: Study Group [file mmc2.docx]

**The COMBAT-AMR study group**

| **First Names** | **Surnames** | **Affiliation** |
| --- | --- | --- |
| Benjamin | Thomas | Port Moresby General Hospital - 3 Mile, Taurama Road National Capital District, Port Moresby, Papua New Guinea |
| Cassius | Maingu | Port Moresby General Hospital - 3 Mile, Taurama Road National Capital District, Port Moresby, Papua New Guinea |
| Dellyne | Polly | Port Moresby General Hospital - 3 Mile, Taurama Road National Capital District, Port Moresby, Papua New Guinea |
| Hans | Nogua | Port Moresby General Hospital - 3 Mile, Taurama Road National Capital District, Port Moresby, Papua New Guinea |
| Jessica | Mondowa | Port Moresby General Hospital - 3 Mile, Taurama Road National Capital District, Port Moresby, Papua New Guinea |
| Joe | Sokal | Port Moresby General Hospital - 3 Mile, Taurama Road National Capital District, Port Moresby, Papua New Guinea |
| Josen | Yem | Port Moresby General Hospital - 3 Mile, Taurama Road National Capital District, Port Moresby, Papua New Guinea |
| Joyce | Lawrence | Port Moresby General Hospital - 3 Mile, Taurama Road National Capital District, Port Moresby, Papua New Guinea |
| Mathilda | Rarah | Port Moresby General Hospital - 3 Mile, Taurama Road National Capital District, Port Moresby, Papua New Guinea |
| Rose | Olwont | Port Moresby General Hospital - 3 Mile, Taurama Road National Capital District, Port Moresby, Papua New Guinea |
| Rupert | Marcus | Port Moresby General Hospital - 3 Mile, Taurama Road National Capital District, Port Moresby, Papua New Guinea |
| Saberina | Silas | Port Moresby General Hospital - 3 Mile, Taurama Road National Capital District, Port Moresby, Papua New Guinea |
| Stephanie | Kialo-Davis | Port Moresby General Hospital - 3 Mile, Taurama Road National Capital District, Port Moresby, Papua New Guinea |
| Alison | Macintyre | Nossal Institute for Global Health, Melbourne School of Population and Global Health, The University of Melbourne, 32 Lincoln Square North, Carlton, Australia |
| Philip | Russo | Nursing and Midwifery, Monash University, 47-49 Moorooduc Highway, Frankston, Australia  Department of Nursing Research, Cabrini Health, 154 Wattletree Road, Malvern, Australia |
| Rosaleen | Kehoe | Department of Infectious Diseases, The Alfred Hospital and School of Translational Medicine, Monash University, 85 Commercial Road, Melbourne, Australia |
